# Gambling on an empty stomach: Hunger modulates preferences for learned but not described risks

**DOI:** 10.1002/brb3.2978

**Published:** 2023-04-05

**Authors:** Maaike M. H. van Swieten, Rafal Bogacz, Sanjay G. Manohar

**Affiliations:** ^1^ Nuffield Department of Clinical Neuroscience University of Oxford Oxford UK

**Keywords:** behavioral paradigms, computational modeling, experimental psychology, hunger, neuroscience, risk‐taking

## Abstract

**Introduction:**

We assess risks differently when they are explicitly described, compared to when we learn directly from experience, suggesting dissociable decision‐making systems. Our needs, such as hunger, could globally affect our risk preferences, but do they affect described and learned risks equally? On one hand, decision‐making from descriptions is often considered flexible and context sensitive, and might therefore be modulated by metabolic needs. On the other hand, preferences learned through reinforcement might be more strongly coupled to biological drives.

**Method:**

Thirty‐two healthy participants (females: 20, mean age: 25.6 ± 6.5 years) with a normal weight (Body Mass Index: 22.9 ± 3.2 kg/m^2^) were tested in a within‐subjects counterbalanced, randomized crossover design for the effects of hunger on two separate risk‐taking tasks. We asked participants to choose between two options with different risks to obtain monetary outcomes. In one task, the outcome probabilities were described numerically, whereas in a second task, they were learned.

**Result:**

In agreement with previous studies, we found that rewarding contexts induced risk‐aversion when risks were explicitly described (*F*_1,31_ = 55.01, *p* < .0001, *η*_p_^2^ = .64), but risk‐seeking when they were learned through experience (*F*_1,31_ = 10.28, *p* < .003, *η*_p_^2^ = .25). Crucially, hunger attenuated these contextual biases, but only for learned risks (*F*_1,31_ = 8.38, *p* < .007, *η*_p_^2^ = .21).

**Conclusion:**

The results suggest that our metabolic state determines risk‐taking biases when we lack explicit descriptions.

## Graphical abstract

Our findings are the first account of how hunger differentially modulates decision‐making for experienced and described risks in the same individual. We complement existing results and provide a precise index of preference change, with a formal computational model of how it might arise. This framework may be widely applicable to states of biological need, and reflect a new axis in the neurochemical and affective control of cognition.

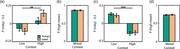

## 1 INTRODUCTION

When we decide between options with uncertain outcomes, we factor risk into the decision. This is most commonly evaluated by asking people to decide between explicitly described, hypothetical choice scenarios (Allais, 1953; Arrow, 1951; Ellsberg, 1961; Kahneman & Tversky, 1979; Weber et al., 2004). In these experiments, risk‐taking is typically modulated by the magnitude and probability of outcomes, or by framing choices in a high‐ or low‐reward context using words or diagrams. This contrasts with real‐life scenarios, in which humans usually make repeated choices, and learn about uncertain outcomes from experience. Several studies have reported that experience‐based choices differ from choices based on verbal or graphical descriptions (Hertwig & Erev, 2009; Hertwig et al., 2004; Niv et al., 2012). This observation is better known as the description–experience gap. In particular, empirical studies have also shown that people are typically risk‐seeking for negatively framed choices, but risk‐averse for positively framed choices when outcomes are explicitly described (Kahneman & Tversky, 1979; Tversky & Kahneman, 1981). However, when choices made from experience are framed in a high‐ or low‐reward context, risk attitudes are reversed compared to description‐based decisions (Hertwig et al., 2004; Ludvig & Spetch, 2011; Ludvig et al., 2014).

The effect of decision context is thought to be driven by anticipatory emotions (De Martino et al., 2006) as well as biological needs (Stephens, 1981). Nevertheless, only a handful of studies has investigated the effect of physiological factors, such as hunger, on risk‐taking from descriptions in humans, and suggest that hunger increases risk‐seeking (Levy et al., 2013; Shabat‐Simon et al., 2018; Symmonds et al., 2010), but the effect of hunger on risk‐taking learned through experience has not yet been tested in humans. Biological need, which is described as the disparity between the current state and the goal state, has been shown to motivate decision‐making in animals that make experiential choices (Aw et al., 2011; Papageorgiou et al., 2016; Pompilio et al., 2006) and has been captured by computational models (van Swieten & Bogacz, 2020). The concept of making decisions to reduce this disparity also underlies the risk‐sensitive foraging theory (Stephens, 1981). According to this theory, if the goal cannot be reached with a safe, low‐risk option, then an individual should choose a high‐risk option because it offers a chance of meeting the need and increases the chance of survival.

The contextual modulation of risk‐taking can be captured by a utility function, such as proposed by the prospect theory for described risks (Kahneman & Tversky, 1979). Recent work has described a model that can account for the contextual modulation of risk‐taking for experienced risks (Moeller et al., 2021). This model is grounded in the theory of dopamine function, because dopamine enhancement promotes risk‐seeking behavior (Gallagher et al., 2007; Rigoli et al., 2016; St. Onge & Floresco, 2009). High‐ or low‐reward contexts may generate a positive or negative prediction error, signaled by dopamine, which might in turn alter risk preference (the Prediction Error Induced Risk‐Seeking [PEIRS] model [Moeller et al., 2021]). Similar to the utility function in prospect theory, PEIRS includes a risk‐sensitivity parameter that determines the impact of context on risk‐taking. Crucially, if hunger alters the extent to which context modulates risk‐taking, this could be captured by changes in this parameter.

Given that experiential and description‐based risk‐taking are thought to involve different neural systems (Fitzgerald et al., 2010), we tested two alternative hypotheses about the effects of hunger on explicitly described versus experientially learned risky choice. On one hand, we might expect the description‐based decision‐making to be modulated by hunger, because risk is tracked and represented in cortical areas that are informed by high‐level cognitive representations, including the prefrontal cortex (Clark et al., 2008; Elliott et al., 1999; Huettel et al., 2005; Knutson & Bossaerts, 2007; St. Onge et al., 2011; Tobler et al., 2007), the parietal (Huettel et al., 2005, 2006), orbitofrontal (Hsu et al., 2005; O'Neill & Schultz, 2010; Tobler et al., 2007), posterior cingulate (McCoy & Platt, 2005), and insular cortex (Knutson & Bossaerts, 2007). It is susceptible to framing effects, whereby the cognitive, numerical, and linguistic context of options influences choice (Allais, 1953; Arrow, 1951; Kahneman & Tversky, 1979) and might therefore be more flexible than the experienced‐based system. Hunger may modulate high‐level decision‐making systems, with the appetite‐stimulating hormone ghrelin activating receptors distributed widely in the cerebral cortex including hippocampus (Zigman et al., 2006) and can enhance memory and performance (Diano et al., 2006). Accordingly, hunger may increase risk‐seeking for explicitly described food but also monetary reward (Levy et al., 2013; Shabat‐Simon et al., 2018; Symmonds et al., 2010), suggesting that metabolic signals could impact cognitive decisions.

On the other hand, we might expect experiential decision‐making to be biased by the organism's needs, because it may rely more on primitive neural systems. The modulation of risk preferences according to energy reserves may be crucial for the adaptation to changes in the environment, in particular when resources are scarce (Houston, 1991; Kacelnik & Bateson, 1997; Stephens, 1981). Experiential decision‐making relies on subcortical brain areas such as the striatum and the dopaminergic midbrain (Abler et al., 2006; Knutson et al., 2001; Niv et al., 2012; Tobler et al., 2007) that are targeted by circulating hormones that signal current energy reserves (Elmquist et al., 1998; Zigman et al., 2006). In particular, leptin inhibits and ghrelin activates dopaminergic neurons in the ventral tegmental area, and could therefore modulate learning and decision‐making via the mesolimbic pathway (Abizaid et al., 2006; Figlewi et al., 2007; Hommel et al., 2006). In line with this, in animal studies, food deprivation increases risk‐seeking in experience‐based tasks (Kacelnik & Bateson, 1997). Perhaps surprisingly, the effects of hunger on experientially learned and explicitly described risk‐taking have never been directly compared.

We employed two risk‐taking tasks in a within‐subject design that have both previously been used to study the involvement of the motivational system (Moeller et al., 2021; Norbury et al., 2013). The described‐risk task involved decisions between two options whose probability of winning and losing, and the magnitude of rewards, was described visually (Rogers et al., 2003). This well‐known task is sensitive to motivation effects (George et al., 2005; Howard et al., 2020; Koester et al., 2013; Norbury et al., 2013; Rock et al., 2013) and specifically, has recently provided evidence that hunger does not affect risk‐taking for described risks (Howard et al., 2020). This task shows outcomes after each decision, contrasting with other tasks from description where hunger increases risk‐taking when outcomes were not provided (Levy et al., 2013; Shabat‐Simon et al., 2018; Symmonds et al., 2010). The learned‐risk task involved decisions between options whose average reward and uncertainty had to be estimated through experience. The presence of outcome feedback for each choice in both tasks means that potential differences in the effects of hunger are driven by how risks are presented, rather than the omission/inclusion of feedback.

Both tasks included three decision contexts, allowing us to verify whether choices were driven by the expected value or by the risk of options. The options presented in a *mixed* context differed in their expected value, which typically drive risk‐neutral behavior. The pair of options in a *low*‐reward context differed in risk, and were matched in expected value, but both options yielded less than the average reward in the task. Options in the *high*‐reward context were analogous to the low‐reward context, but the expected values were both higher than average. These three decision contexts allowed us to examine the effect of both hunger and reward value on risk‐taking learned through experience or risk‐taking for which the risks were described. The described‐risk task achieved this contrast by independently varying both gains and losses. However, to simplify participants’ learning needs in the learned‐risk task, contexts were achieved using only probabilistic *gains* of varying magnitudes. Both tasks also included mixed‐context trials, meaning that the two options had different values.

In agreement with previous studies, we showed that risk attitudes for described risks were opposite to those for learned risks. Hunger only modulated risk preferences for learned risks in a context‐specific manner, showing that the experience‐based system, but not the cognitive system, is sensitive to the motivational drive of an organism.

## 2 METHODS

### 2.1 Participants

Thirty‐two healthy volunteers (females: 20, mean age: 25.6 ± 6.5 years) were recruited for this study. All participants were healthy, had no history of psychiatric diagnoses, had no history of neurological or metabolic illnesses, and had not used recreational drugs in the past 3 months. All participants had a normal weight (Body Mass Index: 22.9 ± 3.2 kg/m^2^), regular eating patterns, and no history of eating disorders. Each participant gave written informed consent and the study was conducted in accordance with the guidelines of the University of Oxford ethics committee. To be able to observe an effect of hunger on risk‐taking, we estimated the effect size from previous papers as 0.25 (Shabat‐Simon et al., 2018; Symmonds et al., 2010). The effect size for detecting an effect of task type on context effects (i.e., the description–experience gap) is 0.6 (Ludvig & Spetch, 2011), which exceeds the effect of hunger on risk‐taking. We designed our study to be able to detect either effect with a power of at least 80%. We used G*Power (3.1.9.7) and estimated that we need 30 participants to obtain a power of 0.85. Post hoc power calculations confirmed that the observed power in our study was 0.8. All data are openly available at https://data.mrc.ox.ac.uk/dataset/effects‐hunger‐experiential‐and‐explicit‐risk‐taking (van Swieten, Manohar, et al., 2021).

### 2.2 Manipulation of metabolic state

**FIGURE 1:**
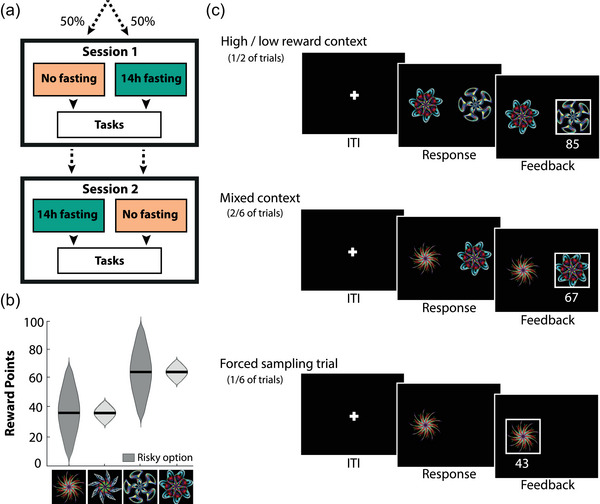
Decisions by experience. (a) Participants were tested in a counterbalanced, randomized crossover design. Participants were tested on two separate days approximately 1 week apart. One session took place after 14 h fasting, the other session after consuming a full meal. (b) Each reward distribution associated with a stimulus was approximately normal. The mean of the distribution was either 35 or 65 with a standard deviation of either 5 or 20. The dark gray distributions indicate the more risky option. (c) Task structure of decisions made from experience. The task consisted of three different trial types: high/low reward context trials (half of the trials), mixed‐context trials (two thirds of the trials), and forced sampling trials (one sixth of the trials). After a response, a reward sampled from the associated reward distribution was presented.

Participants were tested in a within‐subjects counterbalanced, randomized crossover design for the effects of hunger on risk‐taking tasks (Figure 1a). Sessions were approximately 1 week apart (at least 4 days, but no more than 14 days). All sessions took place at the same time of day between 10:00 a.m. and 1:00 p.m., to minimize time‐of‐day effects. For one session, participants were asked to refrain from eating and drinking caloric drinks from 8:00 p.m. the night prior to testing. For the other session, participants were asked to eat normally the day before and consume a full breakfast within 1 h of arriving at the lab for testing. We assessed the effect of fasting on self‐reported feelings of hunger and mood using a computerized Visual Analogue Scale at the start and end of each session (Bond & Lader, 1974; Flint et al., 2000). Participants were asked to place a cursor on a 100‐mm scale with positive or negative text ratings anchored at either end. This assessment provided a subjective measure of whether the manipulation worked. Participants performed the decision‐by‐description task first, then a learning, attention, and planning task not described in this paper (van Swieten, 2020; van Swieten, Bogacz, et al., 2021), and finished with the decision‐by‐experience task. This order was fixed to control for fasting time. Hunger ratings did not significantly change during the fasted session (Wilcoxon signed rank test: *Z* = 1.95, *p* > .05) but increased during the sated session (Wilcoxon signed rank test: *Z* = 4.01, *p* < .0001), indicating that the effect of hunger would be greater for the decision‐by‐description task. Finally, the session order did not affect performance.

### 2.3 Experimental design

#### 2.3.1 Decisions by experience

We employed a modified version of a risk‐taking task developed by Moeller et al. (2021). Participants learned the reward value of four stimuli through repeated sampling. Each stimulus was associated with a Gaussian reward distribution that followed a 2 × 2 design: high or low mean value (65 or 35 points) and high or low standard deviation (20 or 5) (Figure 1b). When a stimulus was chosen, participants received a reward drawn from the corresponding distribution. The task included three trial types: high/low‐reward context trials (50%), mixed‐context trials (33%), and forced sampling trials (17%) (Figure 1c). High‐context trials and low‐context trials consisted of two options with equal mean, but different risks. High‐context trials have a mean above the average outcome in the task. In contrast, low‐context trials have a mean below the average outcome in the task. Mixed‐context trials offered choices between options with unequal expected value. These trials acted as a positive control to check participants paid attention to their choices and understood the difference between the stimuli. Forced sampling trials were trials in which only one stimulus was presented. These trials ensured that all options were sampled from and that participants occasionally experienced reward contingencies that they did not prefer.

Each trial had the same structure. After a short intertrial interval (ITI) of 500–700 ms, the stimuli were presented on the screen. Responses were made by pressing on the left or right arrow key of the keyboard to choose the left or right option, respectively. Choices were immediately followed by feedback for 1.5 s, showing the number of points won (Figure 1c). The total accumulated points were continuously displayed at the top of the screen. Participants were instructed to maximize their total number of points, which was converted into a monetary performance bonus at the end of the task. Each participant completed four blocks of 72 trials. All trial types were equally distributed over the blocks, but we ensured that a stimulus presented in a forced sampling trial did not precede a high/low‐reward context trial with the same stimulus to avoid priming of choices. Reward distributions were generated at the start of each block to ensure each block had the intended reward distribution and stimulus sets were reset after two blocks (or 144 trials). After each block, participants were asked to indicate the reward distribution of each stimulus by placing two cursors on a Visual Analogue Scale ranging from 0 to 100 points, one for the minimum and one for the maximum reward in the distribution. The rated spread was computed as the difference between the rated minimum and maximum of the reward distribution and the rated mean was taken as the average of the two values.

#### 2.3.2 Decisions by description

Risk‐taking behavior from descriptions was probed using the probabilistic task described by Rogers and colleagues (Norbury et al., 2013; Rogers et al., 2003). This task used two trial types—high/low‐reward context trials (one fifth of the trials) and mixed‐context trials (four fifths of the trials). In contrast to the paradigm for decisions by experience, no sampling trials were included in this task since decisions were made based on description and not experience. There were 10 unique gambles; the type of gamble was determined by the probability of winning, the amount that could be won, and the amount that could be lost.

**FIGURE 2:**
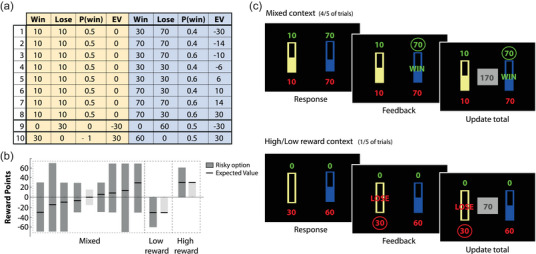
Decisions by description. Decisions made from description were probed using 10 unique gambles, divided into two types of gambles: mixed‐context trials and low/high‐reward context trials. Mixed‐context trials consisted of eight different combinations of points to win, points to lose, and the probabilities of winning, with the expected value of the high‐risk option between −30 to 30 (see gambles 1–8 in panel A), while the low‐risk option always had an expected value of 0. In high/low‐reward context, the expected value of the high‐ and low‐risk option was matched (see gambles 9 and 10 in panel A). (B) Graphical representation of the minimum and maximum reward points for each option. The bold black line indicates the expected value of the option, taking into account the probability of winning, and the points to win and lose. The dark gray distributions indicate the high‐risk option. (C) Task structure of decisions made from description. The task consisted of two different trial types: high/low‐reward context trials (one fifth of the trials) and mixed‐context trials (four fifths of the trials). Each trial consisted of a choice between a low‐risk gamble (yellow) and high‐risk gamble (blue). Points to win and lose were presented in green and red, respectively. The probability of winning corresponded to the size of the filled bar. Feedback was given after each choice and the running total was updated.

High‐ and low‐reward trials offered a choice between a certain win or loss (low‐risk option) and a 50:50 chance gamble (high‐risk option) with the *same expected value*. High‐ and low‐reward gambles were identical in terms of prospect, but differed in valence, allowing for the examination of high‐ and low‐reward context effects on differences in risk attitudes. Mixed‐context trials offered a choice between two options that differed in their objective expected values. The low‐risk option offered a 50:50 chance of winning or losing 10 points, giving an expected value of 0. The other option was a high‐risk gamble, and varied either in the probability of winning (0.6 or 0.4), the magnitude of possible points to win (30 or 70 points), or the magnitude of possible points to lose (30 or 70 points), giving an expected value between −30 and 30 (gambles 1–8 in Figure 2a,b).

The task had the following structure: On each trial, participants were required to choose between two simultaneously presented gambles (Figure 2c). Each gamble was represented visually by a histogram of which the height indicated the relative probability of winning a given number of points. The magnitude of possible points to win was indicated in green above each histogram, with the magnitude of possible points to lose indicated below in red. Visual feedback (win/lose) was given after each choice was made, and the revised running total points was presented before the next trial. Participants were instructed that each gamble should be considered independently of outcomes of previous gambles. Participants completed four blocks of 20 trials, and the order in which gambles were presented was kept constant for both conditions. The highest total score obtained in a block was converted into pence and paid at the end of the task as a performance bonus. Deliberation times were also recorded.

All computerized behavioral paradigms were implemented using Psychophysics Toolbox Version 3 on MATLAB (version 19b; MathWorks, Natick, MA).

### 2.4 Behavioral analyses

Risk was defined as the uncertainty in possible outcomes of a decision, expressed as the variance of the associated reward distribution (Rothschild & Stiglitz, 1970). Risk attitudes were computed separately for high‐ and low‐reward contexts.

For learned risks, the risk preference was averaged over the second half of the trials (72 trials) of each stimulus set (Figure S1A). Using only the second half of the trials allowed participants sufficient opportunity to learn the outcomes associated with each option, while providing a long enough sample to get a reliable measure of their risk preference (Ludvig et al., 2014; Niv et al., 2012).

For described risks, the risk preference was assessed as the proportion of risky gambles chosen in the low (decision type 9) or high (decision type 10) reward context. All trials were included, because no learning occurred and each gamble was considered independently (Figure S1B).

We used the performance on mixed‐context trials as a control measure to verify if people maximized their outcome. The proportion of options with the highest expected value was calculated based on the performance on mixed‐context trials in experienced‐based risk‐taking task and the gambles 1−8 in the description‐based risk‐taking task.

Statistical significance was tested using paired *t*‐tests or repeated measures analysis of variance (ANOVA) as appropriate in MATLAB and SPSS (IBM Corp. Released 2019. IBM SPSS statistics for Windows, Version 26.0. Armonk, NY: IBM Corp.).

### 2.5 Computational model fitting

We used a reinforcement learning model to further assess the effects of hunger on experience‐based risk‐taking. The model itself is described in the results section. We used a hierarchical model‐fitting strategy that takes into account the likelihood of individual participant choices given the individual participant parameters and also the likelihood of the individual participant parameters given the parameter distribution in the overall population across conditions. This two‐stage hierarchical procedure is an estimation strategy of the iterative expectation–maximization algorithm (EM) (Guitart‐Masip et al., 2011; Huys et al., 2012; MacKay, 2003). This regularizes individual participants’ parameter fits, rendering them more robust toward overfitting. To infer the maximum‐a‐posteriori estimate of each parameter for each participant, we set the prior distribution to the maximum‐likelihood given the data of all participants and then use EM for the two conditions separately to obtain parameter estimates for each condition. The statistical significance was tested using paired *t*‐tests with respect to the Gaussian scaled model parameters (see Supporting Information for the transformation of parameters). Reported *p*‐values were corrected for multiple comparisons using the Bonferroni method.

In the fitting procedure, all context trials were used to estimate all parameters. Forced sampling trials were only included for the estimation of learning rates for the mean and variance of a stimulus using Equations (1) and (3), respectively. Due to the absence of a choice, forced sampling trials were excluded from the estimation of the softmax choice parameter and the risk parameters. The presence of only one stimulus makes the probability of choosing this stimulus 1, and this would interfere with the parameter estimation. Initial values for *Q* and *S* were set to 50 and 5, respectively. The model comparison and parameter recovery method can be found in the Supporting Information.

## 3 RESULTS

As expected, participants rated their subjective feelings of hunger significantly higher after 14 h of fasting than after eating a full meal (Wilcoxon signed rank test: *Z* = −4.84, *p* < .0001, *d* = .86), indicating that the manipulation was successful.

### 3.1 Hunger altered experiential risk‐taking in a context‐specific manner

**FIGURE 3:**
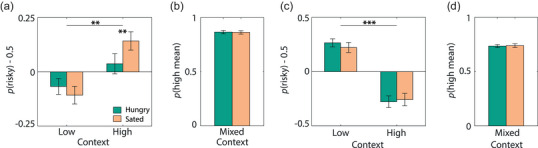
Risk attitudes for learned and described risks. (a) For learned risks, participants were risk‐averse for low‐reward contexts and risk‐seeking for high‐reward contexts. Hunger attenuated risk attitudes for decision contexts in opposite direction. Data are presented with respect to chance level. (b) Proportion of high‐mean options chosen for mixed‐context trials in decisions from experience. (c) For described risks, participants were risk‐seeking for low‐reward contexts and risk‐averse for high‐reward contexts (gambles 9 and 10; Figure 2a). Hunger did not affect these risk preferences. Data are presented with respect to chance level. (d) Proportion of high‐mean options chosen for mixed‐context trials (gambles 1−8; Figure 2a) in described‐risk task. Error bars represent SEM. **p < .01; ***p < .001.

We first analyzed choice behavior in the low‐ and high‐reward context to evaluate experience‐based risk‐taking in a context‐specific manner (Figure 3A). Participants were significantly more likely to choose the risky option in a high‐reward context, but not a low‐reward context (main effect of context [*F*_1,31_ = 10.28, *p* < .003, *η*_p_^2^ = .25]). Such risk‐seeking for high reward and risk avoidance for low‐reward contexts is consistent with previously reported risk attitudes for learned risks (Ludvig et al., 2014; Madan et al., 2015). Crucially, hunger modulated risk‐attitudes for high‐ and low‐reward contexts in opposite manner (interaction effect of hunger and context [*F*_1,31_ = 8.38, *p* < .007, *η*_p_^2^ = .21]), such that hunger neutralized the risk preferences in both contexts. A post hoc paired *t*‐test revealed that this interaction effect was mainly driven by hunger decreasing risk‐taking behavior in the high‐reward context (*t*_31_ = 2.73, *p* = .010, *d* = .49), and not by an increase in risk‐seeking in the low‐reward context (*t*_31_ = 1.01, *p* = .319, *d* = .18).

Although the interaction is significant at a group level, we further asked whether the effect is strong enough to be seen within individuals. For each participant, we ran a post hoc context × hunger logistic regression (Figure S2). Ten out of 32 people had effects that reached significance in the expected direction even within single participants. Only one person had a significant effect in the opposite direction. Finally, hunger did not alter overall risk‐taking behavior (main effect of hunger [*F*_1,31_ = 1.19, *p* = .283, *η*_p_^2^ = .04]).

To verify that neutral risk preferences were not caused by an inability to differentiate stimuli, we used mixed‐context trials to examine whether participants understood the difference in mean and variance of reward distributions. All participants performed on average above 90% on mixed‐context trials, and no participant performed below 60%, indicating that they understood the distinction between high‐ and low‐mean stimuli. The level of hunger did not affect the accuracy on mixed‐context trials (*t*_31_ = 0.62, *p* = .543; Figure 3b).

Finally, the observed changes in risk preferences following hunger were not the result of changes in attention, as the overall reaction times were consistent across conditions (main effect of hunger [*F*_1,31_ < 1]; Figure S3A).

### 3.2 Hunger did not affect risk‐taking from descriptions

To provide a comparable measure to the context effects in experience‐based risk‐taking, we also analyzed the risk preference for matched mean gambles in high‐ and low‐reward context in description‐based choices (Figure 3c). Participants were risk‐seeking for low‐reward contexts and risk‐averse for high‐reward contexts (main effect of context [*F*_1,31_ = 55.01, *p* < .0001, *η*_p_^2^ = .64]). This risk pattern has been previously described by prospect theory (Kahneman & Tversky, 1979), in which extreme positive outcomes are downweighted. In contrast to learned risks, hunger did *not* alter context‐specific risk attitudes for described risks (interaction effect of hunger and context [*F*_1,31_ = 1.53, *p* = .255, *η*_p_^2^ = .05]) or overall risk‐taking (main effect of hunger [*F*_1,31_ < 1]; Figure 3c). In line with previous reports, the risk attitudes for experience‐ and description‐based risk‐taking were opposite, which confirms the existence of the description–experience gap (Hertwig & Erev, 2009).

Participants chose the option with the highest expected value more often in mixed‐decision contexts, regardless of the level of risk (Figure 3d), showing that the difference in expected value drove choice behavior (Weber et al., 2004). In line with the performance on the experience‐based task, but inconsistent with previous findings (Levy et al., 2013; Symmonds et al., 2010), hunger did not attenuate this effect (*t*_31_ = −0.29, *p* = .776). Despite the absence of a shift in risk preference, hunger increased reaction times for all gambles independently of the decision context (main effect of hunger [*F*_1,31_ = 37.42, *p* < .0001, *η*_p_^2^ = .31]; Figure S3B).

### 3.3 Modeling of risk‐sensitive choice behavior

The previous analyses showed that hunger only altered decision‐making when risks had to be learned. However, the behavioral analyses do not provide insight into what computational process was altered by hunger. Therefore, we employed a computational modeling strategy to account for the integration of a specific reward history triggered by sampling. This strategy allowed us to attribute the effects of hunger to a specific computational process. We applied a reinforcement learning model to explain the behavioral data. We used an adapted version of standard reinforcement learning (RW) (Rescorla & Wagner, 1972) that has recently been proposed to account for contextual risk preferences (PEIRS) (Moeller et al., 2021).

Reinforcement learning models describe the learning process in associative learning when subjects learn from the discrepancy between what is expected to happen and what actually happens. The expected mean value of the chosen stimulus *Q*_c_ was updated using:

(1) $$Q_{c,t+1}=Q_{c,t}+\alpha_{Q}\left(r_{t}-Q_{c,t}\right),$$

where *r_t_* is the reward obtained by choosing a stimulus on trial *t* and *α_Q_* is the learning rate for the mean reward. Decisions in this model were solely based on the expected mean value of the presented stimuli. The utility *U* of stimulus *i* on trial *t* was *U_i,t_* = *Q_i,t_*. The probability of choosing an option was computed using the softmax decision rule:

(2) $$P_{c}=\frac{1}{1+e\left(-\beta \left(U_{c}-Uu\right)\right)},$$

where *U*_c_ and *U*_u_ reflect the utility for the chosen and unchosen options, respectively. The parameter *β* determines the participant's tendency to exploit (i.e., to choose the stimulus with the highest *U* value) or to explore (i.e., to randomly choose a stimulus).

The equations so far provide trial‐by‐trial estimates of the expected mean value of each stimulus, but do not consider the variability in outcomes. The PEIRS model extends standard RW learning by accounting for both the average outcome and the variability, or spread (*S*), in outcomes of an action. It also captures innate risk propensities and assumes that high‐ and low‐reward contexts influence how the spread in reward outcomes affects the subjective utility of an action. The spread in reward outcomes was learned in an analogous manner to *Q*‐values using

(3) $$S_{c,t+1}=S_{c,t}+\alpha_{S}\left(\left|r_{t}-Q_{c,t}\right|-S_{c,t}\right),$$

where *α_S_* is the learning rate for the spread, and *r_t_* − *Q*_c,_ *_t_* is the reward prediction error that captures the deviation of the current outcome from the average outcome, which is compared with the current expected spread in reward outcomes *S*_c,_ *_t_*.

The PEIRS model accounts for how participants differentiate matched mean stimuli based on the spread and captures individual risk propensities. For this model, the utility that was entered into the softmax function (Equation 2) depends on the expected mean reward, the spread in reward outcomes, and the sensitivity to the decision context (i.e., the context effect), in the following way:

(4) $$U_{c,t}=\underset{\mathrm{Expected}\;\mathrm{mean}}{\underset{︸}{Q_{c,t}}}+\underset{\mathrm{Risk}\;\mathrm{propensity}}{\underset{︸}{\gamma_{0}\times S_{c,t}}}+\underset{\mathrm{Context}\;\mathrm{effect}}{\underset{︸}{\gamma_{1}\times \delta_{\mathrm{context}}\times S_{c,t}}},$$

where the parameter *γ*_0_ modulates the risk propensity of an individual and reflects the tonic level of dopamine (Mikhael & Bogacz, 2016). A positive value of *γ*_0_ increases risk‐seeking, because a high variance contributes positively to an option's value, meaning that the high‐spread option is preferred. This effect is reversed when *γ*_0_ < 0. Note that the first two terms in Equation (4) are analogous to the mean‐variance models developed for decisions from description (Boorman & Sallet, 2009; D'Acremont et al., 2009).

The third term captures the biasing effect of high‐ or low‐reward contexts on choice behavior. Context effects play an important modulatory role in risky decision‐making (De Martino et al., 2006; Tversky & Kahneman, 1981) and were also observed in the current study. The context reflects how the expected value of the presented stimuli compares to the overall expected value of all stimuli in the task $\delta_{\mathrm{context}}=Q_{\mathrm{presented},t}-Q_{\mathrm{all},t}$, where $Q_{\mathrm{presented},t}$ is the average of the *Q*‐values of the stimuli on the current trial and $Q_{\mathrm{all},t}$ is the average of the *Q*‐values of all four stimuli. The true average value of all stimuli is 50 points, but $Q_{\mathrm{all},t}$ fluctuates around 50 as the *Q*‐values of the stimuli change by trial‐to‐trial updates. High‐reward contexts have an objective value above the average ($\delta_{\mathrm{context}}^{\mathrm{high}}=65-50=+15$ points), whereas low‐reward contexts have a context value below the average ($\delta_{\mathrm{context}}^{\mathrm{low}}=35-50=-15$ points). The parameter *γ*_1_ is a gain parameter that determines the extent to which the decision context and spread in reward outcomes contribute to choice behavior. Positive values of *γ*_1_ increase risk‐taking behavior in high‐reward contexts, and reduce risk‐seeking in low‐reward contexts. The opposite is true for negative values of *γ*_1_. In the PEIRS model, the effects of hunger can be attributed to how participants learn about the expected value (reflected by *α_Q_*), the spread of reward outcomes (reflected by *α_S_*), the individual risk propensity (reflected by *γ*_0_), and/or sensitivity to the context (reflected by *γ*_1_). For example, if hunger makes people inherently more risky, we would see an increase in the baseline risk propensity *γ*_0_ of the individual. If hunger influences risk‐taking dependent on the context the choices are presented in, we would see a change in *γ*_1_. If hunger influences how individuals learn about the mean and variance of outcomes, we would see a change in *α_Q_* and *α_S_*, respectively. A slower learning rate could contribute to an under‐/overvaluation of the mean and/or variance of a stimulus, since the individual may not have had enough time or exposure to learn the “true” reward value.

### 3.4 Computational modeling captured risk preferences

**FIGURE 4:**
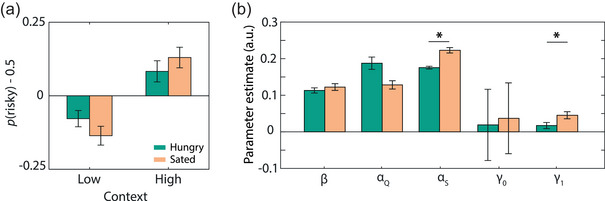
Model fitting results with the Prediction Error Induced Risk‐Seeking (PEIRS) model. (a) Simulated choice behavior using estimated parameters for the fasted and sated condition. Simulated data showed a similar pattern to the behavioral data depicted in Figure 3a. Data are presented with respect to chance level. (b) Hunger significantly decreased the learning rate for reward spread *α_S_*, and the sensitivity to contexts *γ*_1_. Hunger did not alter the softmax temperature *β*, the learning rate for mean *α_Q_*, or individual risk preferences *γ*_0_. Error bars represent SEM. Plotted parameters are the bounded model parameters. Statistical significance was tested with respect to the unconstrained Gaussian distributed parameters. *p < .05.

To confirm that the PEIRS model described risk preferences, we compared it to a simplified model without *γ* parameters, that is, a simple Rescorla–Wagner model. Over 70% (23 out of 32 participants) were better described by the PEIRS model (BIC_RW_ = 16,915 and BIC_PEIRS_ = 15,996), confirming that the addition of extra parameters was justified. The quality of the fitting procedure was verified with a parameter recovery analysis. All parameters were well recovered (.75 < *R* < .95) and the model fitting procedure did not introduce spurious correlations between the other parameters (|*R*| < .3; Figure S4). Surrogate data generated with the best fitted parameters specifically confirmed that the model reproduces the key effect of hunger on choice preferences (Figure 4a).

In line with the behavioral analyses, we found an effect of hunger on parameter estimates obtained with the PEIRS model (Figure 4b). On average, hungry participants had lower learning rates for the spread (*α_S_*, *p* < .0001, *d* = .70) and a lower sensitivity to context effects (*γ*_1_, *p* = .02, *d* = .55), making them risk neutral across decision contexts. Hunger did not significantly alter learning rates of mean values (*α_Q_*, *p* = .165, *d* = .48) or choice stochasticity (*β*, *p* = 1, *d* = .13). Although risk propensities were differently affected by hunger among individuals, at the group level, individual risk propensities were not significantly altered by hunger (*γ*_0_, *p* = 1, *d* = .04).

### 3.5 Subjective rating reflects learned utility

**FIGURE 5:**
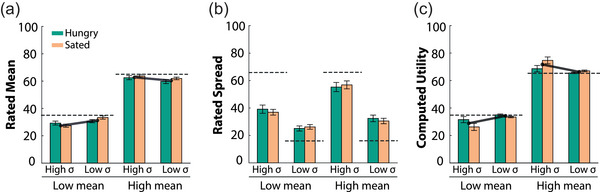
Subjective rating reflects learned utility. At the end of each block, participants indicated the mean (a) and spread (b) of the distribution associated with each stimulus. (c) The computed utility for each of the stimuli (Equation 4) reflects the same pattern as the subjectively rated mean values. Dashed lines indicate objective mean or spread in the reward points. Error bars represent SEM.

We also asked participants to indicate the reward distribution of each stimulus on a Visual Analogue Scale at the end of each block. We used these measures to examine whether people distinguished the stimuli based on the true mean and variance, or a scaled version of the objective values. We found that the subjectively rated mean and spread of each reward distribution (Figure 5a,b) showed a similar pattern as the objective values (Figure 1b).

Participants were able to reliably distinguish stimuli based on their mean (main effect of mean [*F*_1,31_ = 831.91, *p* < .0001, *η*_p_^2^ = .96]; Figure 5a). The average outcome for the risky option was rated higher for high‐mean stimulus, but lower for the low‐mean stimulus (interaction effect of true mean and spread [*F*_1,31_ = 11.19, *p* < .002, *η*_p_^2^ = .27]). To highlight this effect, Figure 5A includes lines connecting the mean ratings of low‐ and high‐variance stimuli, which have different slopes for high‐mean and low‐mean stimuli. These findings are consistent with the risk preferences observed in Figure 3a, showing that participants valued their preferred stimulus more. This effect was less strong in hungry individuals, who rated the mean of stimuli in the high‐ and low‐reward context more similarly, regardless of the level of risk (interaction effect of hunger and spread [*F*_1,31_ = 9.48, *p* < .004, *η*_p_^2^ = .23]). The rated values are in line with the risk‐neutral choice behavior of hungry individuals (Figure 3a).

All participants understood that matched mean stimuli differed in the level of spread (main effect of spread [*F*_1,31_ = 61.93, *p* < .0001]; Figure 5b). However, participants rated the spread for high‐mean options consistently higher than that for low‐mean options (main effect of mean [*F*_1,31_ = 86.56, *p* < .0001]). Furthermore, the perceived contrast in variance for high‐mean options was larger compared to the perceived contrast for low‐mean options (interaction effect mean and spread [*F*_1,31_ = 32.45, *p* < .0001, *η*_p_^2^ = .51]).

Given that we observed biases for the preferred (i.e., most chosen) stimulus in the subjective ratings, we examined whether this was reflected by the learned utility. The utility of each of the stimuli (Equation 4) was computed using the *Q* and *S*‐values obtained from the simulations in Figure 4a and the best fitted parameters of each individual. During the ratings, only one stimulus is presented at the time, thus $\delta_{\mathrm{context}}=Q_{\mathrm{rated}\mathrm{stimulus}}-\mathrm{mean}\left(Q_{\mathrm{all}\mathrm{stimuli}}\right)$. The utilities were computed for each stimulus set separately and averaged across individuals (Figure 5c). We observed three analogous effects in the learned utility as observed in the subjectively rated mean values (Figure 5a vs. Figure 5c). First, the utility of high‐mean stimuli was significantly higher than the utility of low‐mean stimuli (main effect of mean [*F*_1,31_ = 319.85, *p* < .0001, *η*_p_^2^ = .91]). Second, the learned utility for the risky option was higher for the high‐mean stimulus, but lower for the low‐mean stimulus (interaction effect of mean and variance [*F*_1,31_ = 19.32, *p* < .0001, *η*_p_^2^ = .38]). Third, hunger altered the learned utility. Hunger increased the utility for low‐mean stimuli, but not for high‐mean stimuli (interaction effect of mean and hunger [*F*_1,31_ = 6.21, *p* = .018, *η*_p_^2^ = .17]). This effect was specific for high‐variance options, but not low‐variance options (interaction effect of mean, variance, and hunger [*F*_1,31_ = 5.86, *p* = .022, *η*_p_^2^ = .16]).

## 4 DISCUSSION

Using information about the current metabolic state to adapt to variable reward outcomes is critical for survival (Stephens, 1981). In this study, we used two tasks to test whether hunger selectively affected risk‐taking for learned or explicitly described options. We found that hunger modulated risk attitudes for decisions whose outcome statistics had to be learned, but not for decisions whose outcome statistics were explicitly described. Furthermore, hunger promoted risk aversion for high‐reward contexts, but not for low‐reward contexts. These results suggest that the current metabolic state drives adaptive behavior for trial‐and‐error learning in a context‐specific manner, but may not alter the integration of factual information.

**FIGURE 6:**
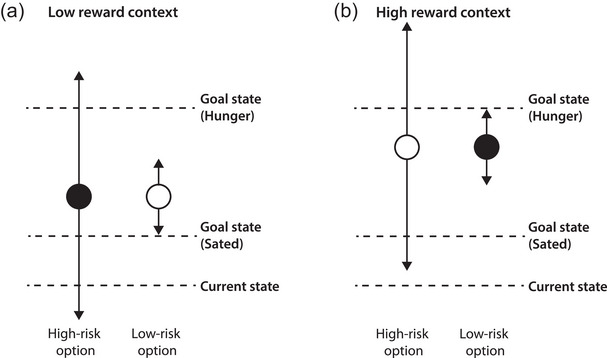
“Optimal” choice scenarios for high‐ and low‐reward contexts. The circles denote the expected value of the high‐ and low‐risk option and the arrows denote the spread of the reward. Filled circle indicates the preferred choice. Panel (a) represents a scenario in a low‐mean/low‐reward context. When forced to choose between options of similar expected value but different risk (i.e., outcome variance), decision‐makers should prefer high‐risk options (filled circle) when hungry (because it is the only option that offers a chance of fulfilling their need), and prefer low‐risk options (open circle) when sated to ensure the goal state is achieved and avoid unnecessary downside costs that might be incurred if the high‐risk option is chosen. Panel (b) represents a scenario in a high‐mean/high‐reward context. The goal state can now be achieved with the low‐risk option so this should be chosen in a high‐reward context. The risky option should only be chosen if the needs cannot be met by choosing a safe option. Sated individuals can afford the costs (as this is still close to their goal state) and may therefore be more willing to gamble.

As postulated by the risk‐sensitive foraging theory (Stephens, 1981), individuals should make decisions that minimize the disparity between the goal and the current state to maximize the chance of survival. When forced to choose between two low‐reward options of similar expected value but different risk, the high‐variance option should be preferred when hungry, because this is the only option that offers a chance of fulfilling the current biological need (Figure 6a). In contrast, when higher rewards are at stake, hungry individuals should now opt for the low‐risk option, because this option allows them to fulfil their need, without incurring an unnecessary cost that may compromise survival (Figure 6b). Although the participants in this study were not starving and the rewards in this task may only indirectly (via money) fulfil their biological needs, we found shifts in risk preferences (Figure 3a) that follow the predictions by the risk foraging theory as explained in Figure 6. Our data illustrate that hunger has the tendency to alter risk‐taking in line with important evolutionary processes from the past.

While risk‐taking from description and experience are both modulated by the contextual value of the options presented, they are not equally susceptible to modulation by hunger. For experience‐based decisions, information about the availability of reward and the metabolic need is integrated (Abizaid et al., 2006; Aitken et al., 2016; Cone et al., 2016; Hommel et al., 2006; Papageorgiou et al., 2016), whereas the evaluation of description‐based decisions is susceptible to reward availability only.

Importantly, the behavioral data showed that the decision context was important for choice behavior. For example, participants preferred the risky option in high‐reward contexts, but preferred the safer option when it was presented with a low‐mean stimulus in a mixed context. This contextual adaptability is beneficial for survival and recent work has provided a mechanistic explanation for these contextual effects in experiential risk‐taking (Moeller et al., 2021). Pupil dilation at the time of decision context tracked how surprising the context was, corresponding to $|\delta_{\mathrm{context}}|$. Furthermore, across individuals this dilation independently correlated with the size of *γ*_1_, which controls how strongly the context biases choices. Crucially, in the present study, the effects of hunger were directly reflected by this parameter. Sated individuals showed a different choice bias in each decision context, while hungry participants were risk neutral across both decision contexts. Hunger has been previously associated with maladaptive behavior (Bartholdy et al., 2016; Kirk & Logue, 1997; Skrynka & Vincent, 2019); however, the results in this study show that hunger makes people more “rational” in their behavior. These individuals rely more on the objective expected value of an option, rather than the subjective expected utility (von Neumann & Morgenstern, 1944).

What might the cognitive mechanisms of the hunger effect be? One possibility is that hunger might increase cognitive load or reduce memory capacity. These two domains might be more important for decisions from experience than decisions made from descriptions. Memory biases, particularly for big wins, could contribute to the asymmetrical effects we observe for experienced risk (Madan et al., 2017). It is also possible that our results stem from using monetary, secondary reward, rather than primary rewards like food, which have been more extensively used in the eating behavior literature (De Araujo et al., 2020; Murray et al., 2014). Future work could compare contextual effects of abstract rewards with primary food rewards.

Hunger did not affect description‐based risk‐taking, which is in line with the findings by Howard et al. (2020). However, this result may be surprising when compared to earlier studies that reported increased risk‐seeking for food, water, and monetary rewards when gambles were explicitly described to hungry individuals (Levy et al., 2013; Shabat‐Simon et al., 2018; Symmonds et al., 2010). One of the obvious differences between the studies with and without an effect is the presence of feedback. Studies involving described risks typically omit feedback. This approach may be acceptable for a laboratory setting; real‐world choices usually lead to outcomes even if the outcome probabilities are known. Although feedback about described risks could alter risk attitudes (Jessup & O'Doherty, 2010), we did not find evidence that learning occurred in this task as there was no change in risk preferences over the course of the task or across sessions. In addition to the presence of feedback, there are two additional differences in task design that may contribute to the observed effect. First, previous studies mostly concerned a decision between a fixed certain amount and a risky alternative (Levy et al., 2013; Shabat‐Simon et al., 2018), whereas the current study compared two risky options (as in Symmonds et al., 2010), so one possibility is that hunger affects how risk is compared against certainty. Second, our task included 10 unique choice types that were played eight times each, which might increase familiarity and promote explicit rational processing; in contrast, previous studies used trial‐unique gambles that were only played once.

One potentially relevant difference between the two tasks in the present study is the proportion of mixed‐context (easy) trials; in the description task, half of trials were mixed context, whereas in the experience task, they were in a 4:1 ratio. This was primarily to keep the description‐based task matched to previous work. Further, it allowed us to look for any hunger‐related differences between loss, gain, and probability processing, which were absent in our data. We have not run this task without losses so we cannot be certain the effects would hold with rewards only.

One might also argue that the differential effect of hunger on risk‐taking could be driven by the strong effect of task type. We observe that the overall risk‐taking behavior in both tasks is neutral, which is illustrated by an overall *p*(risky) of .5 and a *γ*_0_ around 0. The direction of the context effect also reverses as a result of our modulation. Therefore, we believe that the effect of hunger on risk taking is not attributable to a main effect of task type. We also considered whether the differential results could be caused by the order the tasks were administered in, rather than the type of task. We believe that the experience in one task is not likely to affect the other task, since the tasks are fundamentally different, they were not introduced to the participants as gambling tasks, and participants were unaware that these tasks would be compared later. Possible order effects could have altered baseline risk preferences. However, the order of the tasks was fixed, making it therefore unlikely that the effect of hunger on risk preference would be affected as a result.

As in previous studies (e.g., Clark et al., 2008; Fitzgerald et al., 2010; Madan et al., 2017), the decisions from description contained exact repeated trials. On one hand, this matches the experience task, where the four options and their symbols were fixed. On the other hand, decisions from experience arguably have different values on each trial due to learning. This could lead to discrepancies between the tasks, but it is not easy to match description and experience tasks exactly in this respect.

We first opted for a design that was more similar in reward outcomes to the experience‐based task (similar to the design by Symmonds et al., 2010), but a pilot study showed that using normally distributed reward outcomes (instead of discrete rewards as used by Symmonds et al.) complicated the task and failed to induce clear risk preferences. We are also not aware of any established risk‐taking by description task design that includes rewards that are drawn from a normal distribution. We therefore opted for a task that has been previously used to measure changes in risk preferences following the manipulation of the dopamine (motivational) system (Norbury et al., 2013).

Our data suggest that hunger does not impact risk‐taking for description‐based choices, at least when explicitly comparing two risky options with feedback provided, perhaps because the neural processes that drive explicit risk‐taking are not under the direct control of hunger.

An important contribution of the current study is that we compared the effect of hunger on risk preferences for description‐ and experience‐based risks in the same individual following the same level of deprivation. It may be difficult to compare our results to those of existing studies due to varying levels of deprivation; some studies report 4 h of fasting (Levy et al., 2013), others report 12 h (Shabat‐Simon et al., 2018; Symmonds et al., 2010) or even 20 h of fasting (Howard et al., 2020). Furthermore, risk preferences vary greatly among individuals (Levy et al., 2013). Previous studies showed that hunger had a converging effect on a population—individuals who were highly risk‐averse when satiated became less averse when hungry, while risk‐seeking individuals became more risk‐averse (Levy et al., 2013).

Our study demonstrates that the opposing risk patterns in the description–experience gap are driven by how risks are presented, rather than individual risk propensities. Previous studies suggested that these different risk patterns arise from memory biases (Madan et al., 2014, 2017) or under‐ and overweighting of rare events in description‐ and experience‐based choices, respectively (Hertwig, 2012; Hertwig et al., 2004; Kahneman & Tversky, 1979). The dissociable effect of hunger on experiential and explicit risk‐taking in this study suggests that the neural processes driving these preferences are, at least partially, distinct (Fitzgerald et al., 2010; Jessup & O'Doherty, 2010).

In conclusion, we found that hunger decreased risk‐taking for high‐reward context in decisions where outcome statistics had to be learned. This observation matches optimal foraging theory, which predicts a survival advantage when individuals consider the variability of resources in the environment according to the current level of energy reserves. For learned risks, hungry individuals considered their metabolic need and the availability of rewards when making choices, whereas sated individuals only considered the availability for rewards. Hunger did not alter explicit risk‐taking, suggesting that cognitive evaluation of risk may be unaffected. This is the first study that uses a within‐subject design to test the effects of hunger on risk attitudes for decisions involving learned and described risks in high‐ and low‐reward contexts. It provides new insights into the modulatory role of hunger in adaptive behavior. Further studies will need to address the neural processing involved in the effects of hunger on decision‐making under uncertainty.

### PEER REVIEW

The peer review history for this article is available at https://publons.com/publon/10.1002/brb3.2978.

## Supporting information

Supporting Information

Supporting Information

Supporting Information

Supporting Information

**Figure S1**: Learning occurs in experienced‐based risk‐taking, not in description‐based risk‐taking.
**Figure S2**: Pronounced context effects in a third of the participants.
**Figure S3**: Reaction times for experienced and described risk.
**Figure S4**: Parameter recovery for the PEIRS model.

## Data Availability

All data are openly available at https://data.mrc.ox.ac.uk/data‐set/effects‐hunger‐experiential‐and‐explicit‐risk‐taking (van Swieten, Manohar, et al., 2021).
