# Supplementary material for: Gambling on an empty stomach: Hunger modulates preferences for learned but not described risks
Source: Brain Behav. 2023 Apr 5;13(5):e2978. doi: 10.1002/brb3.2978 (PMC10176009; doi:10.1002/brb3.2978)
Supplement: Supplementary file 5 — Figure S1: Learning occurs in experienced‐based risk‐taking, not in description‐based risk‐taking. Figure S2: Pronounced context effects in a third of the participants. Figure S3: Reaction times for experienced and described risk. Figure S4: Parameter recovery for the PEIRS model. [file BRB3-13-e2978-s003.docx]

# Supplemental material

## Learning occurred for experienced, but not for described risks


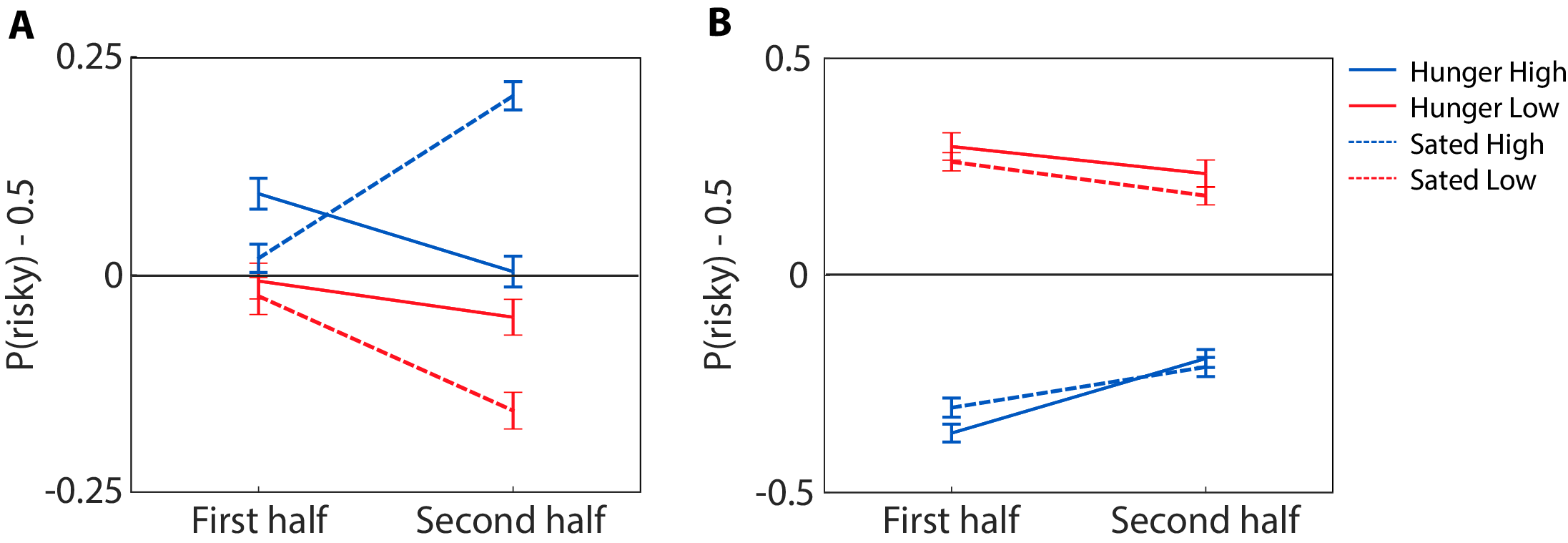


Figure S1: **Learning occurs in experienced-based risk-taking, not in description-based risk-taking.** Mean proportion of risky choices for the high (blue) or low (red) contexts as a function of blocks for the hungry (solid lines) and sated (dotted lines) condition. **A)** In the experience-based task, individuals are more risk-seeking for high reward contexts than for low reward contexts (main effect context [*F*_1_*_,_*_31_ = 8*.*87, *p <* 0*.*006, *η_p_*^2^ = 0*.*22]). Risk preferences change over time, but differently dependent on the decision context (interaction effect of time and context [*F*_1_*_,_*_31_ = 6*.*69, *p* = 0*.*015, *η_p_*^2^ = 0*.*18]), showing that learning occurs in this task. This effect is also modulated by the level of hunger (interaction effect of hunger, context and time [*F*_1_*_,_*_31_ = 6*.*34, *p* = 0*.*017, *η_p_*^2^ = 0*.*17]). **B)** In description-based risk taking, individuals are more risk-seeking for low reward contexts compared to high reward contexts (main effect context [*F*_1_*_,_*_31_ = 55*.*01, *p <* 0*.*0001, *η_p_*^2^ = 0*.*64]). Risk preferences do not change over time or as a result of hunger (all *p*-values *>* 0*.*1). Each bin contains equal number of high and low reward context trials. Error bars represent within-subject SEM.

## Pronounced context effects in a third of the participants


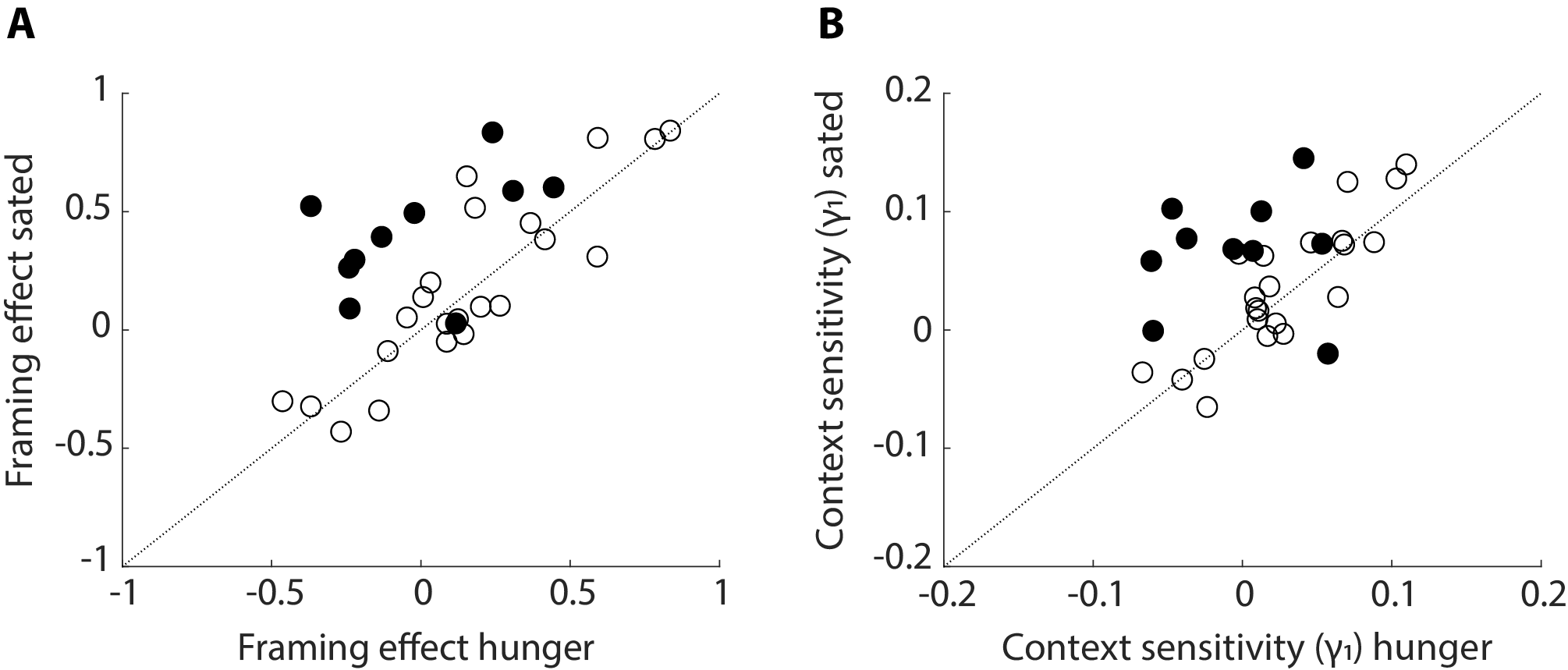


Figure S2: **Pronounced context effects in a third of the participants.** Participants with a significant interaction effect of context × hunger in the logistic regression are represented by filled circles. **A)** Individuals with a significant interaction effect show a greater framing effect when sated. The framing effect is measured as P(risky | high reward context)−P(risky | low reward context). **B)** Individuals with a significant interaction effect showed greater context sensitivity (*γ*_1_ parameter derived from computational modelling) when sated.

## Hunger increased reaction times for described, but not experienced risks


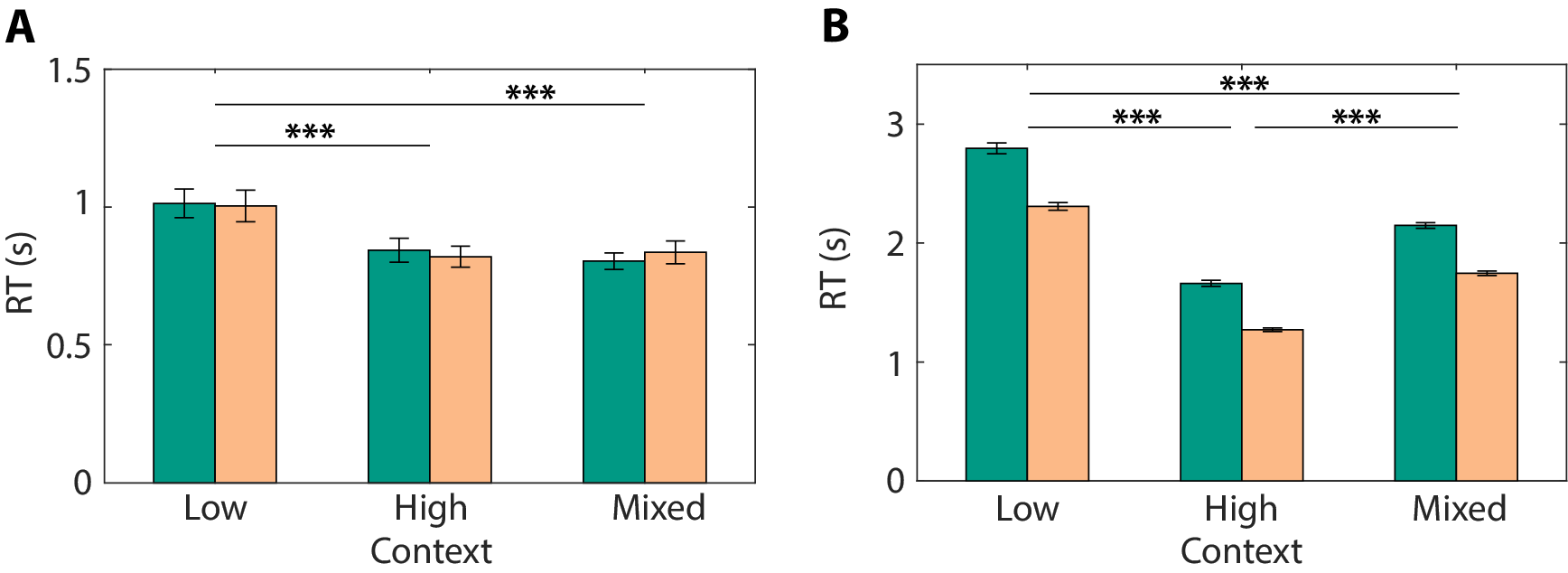


Figure S3: **Reaction times for experienced and described risk. A)** Median reaction times for experience-based risk-taking, split by context type. Hunger did not affect overall reaction times for experienced risks (main effect of hunger [*F*_1_*_,_*_31_ *<* 1]; Suppl. Fig. S3A). Participants’ reaction times did vary depending on the decision context (main effect of decision context [*F*_2_*_,_*_62_ = 37*.*67, *p <* 0*.*0001, *η_p_*^2^ = 0*.*64]). Further analyses using a two-way repeated measures ANOVA revealed that participants took significantly longer to respond for low reward contexts compared to high reward contexts [*F*_1_*_,_*_31_ = 51*.*39, *p <* 0*.*0001, *η_p_*^2^ = 0*.*62] and mixed contexts [*F*_1_*_,_*_31_ = 39*.*19, *p <* 0*.*0001, *η_p_*^2^ = 0*.*56], without showing a significant difference in reaction times between high reward contexts and mixed contexts [*F*_1_*_,_*_31_ = 0*.*55, *p* = 0*.*464]. These data suggest that low reward contexts were perceived as more difficult than high reward or mixed contexts, a finding that has been reported before (Madan et al., 2015). Hunger did not affect context-specific reaction times (interaction effect of hunger and context [*F*_2_*_,_*_62_ *<* 1]). **B)** Median reaction times for description-based risk-taking, split by context type. Hunger increased reaction times for described risks (main effect of hunger [*F*_1_*_,_*_31_ = 37*.*42, *p <* 0*.*0001, *η_p_*^2^ = 0*.*31]; Suppl. Fig. S3B). Participants took significantly longer to respond for low reward contexts compared to high reward contexts [*F*_1_*_,_*_31_ = 61*.*96, *p <* 0*.*0001, *η_p_*^2^ = 0*.*67] and mixed contexts [*F*_1_*_,_*_31_ = 18*.*79, *p <* 0*.*0001, *η_p_*^2^ = 0*.*38]. They also deliberated longer for choices in mixed decision contexts compared to high reward contexts [*F*_1_*_,_*_31_ = 25*.*80, *p <* 0*.*0001, *η_p_*^2^ = 0*.*45]. Error bars represent SEM. *** *p <* 0*.*001.

## Computational modelling

We optimised the model parameters by minimising the negative log-likelihood (LL) of the data, given different parameter settings, using MATLAB’s fminunc function to obtain unconstrained parameter estimates:

LL = log(*P*(data|model)) (1)

### Parameter transformations

The unconstrained Gaussian distributed parameter estimates *x_i_* ∼ N(*µ_x_,σ_x_*), with a population mean of *µ_x_* and a standard deviation of *σ_x_*, were transformed into bounded model parameters using logistic/hyperbolic/exponential transformations to ensure that the model parameters were biologically plausible and interpretable. This transformation is justified by the premise that each individual subject (with a parameter value *x_i_*) is randomly drawn from a population of subjects with normally distributed parameters (with population mean *µ_x_* and a standard deviation of *σ_x_*). This is important for our analysis because normally distributed parameters permit the use of parametric tests to identify differences between conditions. We denote the model parameters by Greek letters and the Gaussian scaled parameter estimates by their respective Latin letters.

The relationship between the [0,1]-bounded model parameter *α* and the Gaussian parameter estimate *a* is given by a logistic function:

*α* = 1*/*(1 + exp(−*a*))*.* (2)

The relationship between the [-1,1]-bounded model parameter *γ*_0_, and *γ*_1_ and the Gaussian parameter estimate *g*0 and *g*1 is given by the hyperbolic tangent function:


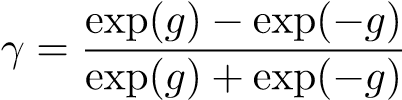
*,* (3)

The relationship between the [0*,*∞)-bounded (logarithmically scaled) model parameter *β* and the Gaussian parameter estimates *b* and *p* are given by the exponential function:

| *β* = exp(*b*)*,* | (4) |
| --- | --- |
| *π* = exp(*p*)*.* | (5) |

### Model comparison

To identify the best fitting model, we compared the models using the Bayesian Information Criterion (BIC) value (Schwarz, 1978), which considers differences in model complexity. The BIC value was calculated as:

*BIC* = 2*L* + *k* ln(*n*)*,* (6)

where *L* is the negative log likelihood, *n* the number of choices and *k* the number of free model parameters. The BIC value was computed using the maximum likelihood function of the population data across conditions.

The model with the lowest BIC values was the best fitting model.

### Parameter recovery

To validate the parameter estimates generated by the fitting procedure, we conducted a parameter recovery analysis. For each parameter, we generated samples from the marginalised posterior distribution of the fit to get realistic parameters that could describe choice behaviour. The generated parameters were uncorrelated (|*R*| *<* 0*.*3), allowing for testing whether the fitting procedure introduced any confounding factors. We used the generated sets of parameters to simulate choice behaviour and used a hierarchical model fitting procedure to estimate parameters for the simulated data (“Recovered parameters”). We then assessed the quality of the parameter recovery by comparing the true parameters used to simulate data with the recovered parameters. We calculated the Pearson correlation between all pairs of recovered parameters to test whether the fitting procedure introduced spurious correlations. A strong correlation between the true and recovered parameters indicates a good recovery of the parameters and reliable model fitting results. The quality of the fitting procedure was verified with a parameter recovery analysis. All parameters were well recovered (0*.*75 *< R <* 0*.*95) and the model fitting procedure did not introduce spurious correlations between the other parameters (|*R*| *<* 0*.*3; Fig. S4).


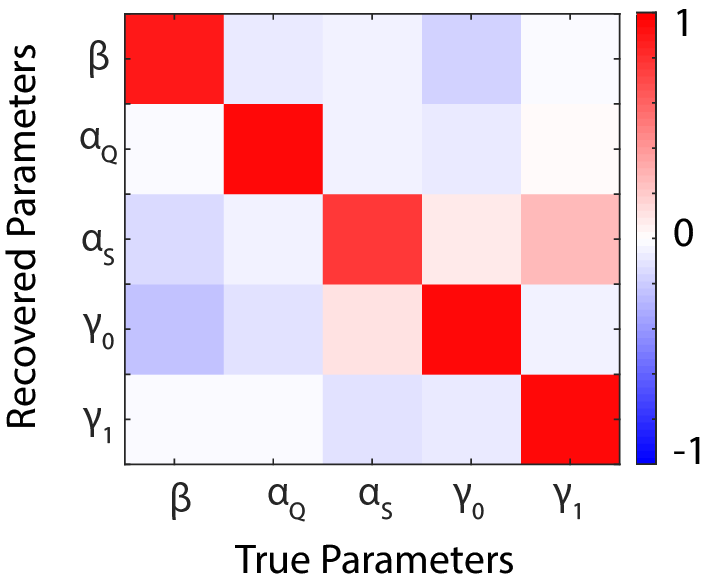


Figure S4: **Parameter recovery for the PEIRS model.** Correlation matrix of the free parameters used to generate the simulated data (‘True parameters’) and the obtained parameters by applying the parameter estimation procedure on the simulated data (‘Recovered parameters’). Bright red values indicate a strong correlation between the true and recovered parameter value and therefore a good parameter recovery.

# References

Madan, C. R., Spetch, M. L., and Ludvig, E. A. (2015). Rapid makes risky: Time pressure increases risk seeking in decisions from experience. *Journal of Cognitive Neuroscience*, 27(8):921–928.

Schwarz, G. (1978). Estimating the dimension of a model. *Annals of Statistics*, 6(3):461–464.
